# DREADD Activation of Pedunculopontine Cholinergic Neurons Reverses Motor Deficits and Restores Striatal Dopamine Signaling in Parkinsonian Rats

**DOI:** 10.1007/s13311-019-00830-4

**Published:** 2020-01-21

**Authors:** Puneet K. Sharma, Lisa Wells, Gaia Rizzo, Joanna L. Elson, Jan Passchier, Eugenii A. Rabiner, Roger N. Gunn, David T. Dexter, Ilse S. Pienaar

**Affiliations:** 1 Centre for Neuroinflammation and Neurodegeneration, Division of Brain Sciences, Faculty of Medicine, Imperial College London, London, W12 0NN UK; 2 Invicro, Hammersmith Hospital Campus, Imperial College London, London, W12 0NN UK; 3 Institute of Genetic Medicine, Newcastle University, Newcastle upon Tyne, NE1 3BZ UK; 4 School of Life Sciences, University of Sussex, Falmer, BN1 9PH UK

**Keywords:** Cholinergic, dopamine, DREADD, motor behavior, pedunculopontine nucleus, positron emission tomography.

## Abstract

The brainstem-based pedunculopontine nucleus (PPN) traditionally associates with motor function, but undergoes extensive degeneration during Parkinson’s disease (PD), which correlates with axial motor deficits. PPN-deep brain stimulation (DBS) can alleviate certain symptoms, but its mechanism(s) of action remains unknown. We previously characterized rats hemi-intranigrally injected with the proteasomal inhibitor lactacystin, as an accurate preclinical model of PD. Here we used a combination of chemogenetics with positron emission tomography imaging for *in vivo* interrogation of discrete neural networks in this rat model of PD. Stimulation of excitatory designer receptors exclusively activated by designer drugs expressed within PPN cholinergic neurons activated residual nigrostriatal dopaminergic neurons to produce profound motor recovery, which correlated with striatal dopamine efflux as well as restored dopamine receptor 1- and dopamine receptor 2-based medium spiny neuron activity, as was ascertained with c-Fos-based immunohistochemistry and stereological cell counts. By revealing that the improved axial-related motor functions seen in PD patients receiving PPN-DBS may be due to stimulation of remaining PPN cholinergic neurons interacting with dopaminergic ones in both the substantia nigra pars compacta and the striatum, our data strongly favor the PPN cholinergic–midbrain dopaminergic connectome as mechanism for PPN-DBS’s therapeutic effects. These findings have implications for refining PPN-DBS as a promising treatment modality available to PD patients.

**Electronic supplementary material:**

The online version of this article (10.1007/s13311-019-00830-4) contains supplementary material, which is available to authorized users.

## Introduction

Targeting of different brain regions using deep brain stimulation (DBS), as a therapeutic option available to Parkinson’s disease (PD) patients, has been successfully used as a surgical approach to treat both the symptoms (e.g., tremor) and also the side effects of dopamine (DA) replacement strategies (e.g., dyskinesias) for more than 2 decades [1]. Low-frequency DBS can be targeted to the pedunculopontine nucleus (PPN), leading to an improvement in motor-related symptoms, particularly gait and postural abnormalities, which affect a significant number of PD patients [2–5]. The PPN, located in the dorso-lateral mesopontine tegmentum, is a highly heterogeneous neuronal structure, primarily composing of cholinergic, glutamatergic, and gamma-aminobutyric acid (GABA)ergic neurons [6–13]. Clinical interest in the PPN as a DBS target stems from the partial denervation of PPN cholinergic neurons (where in the intact brain, such neurons innervate the basal ganglia motor loop [9, 14]) observed in postmortem brains of PD patients [9, 15, 16]. The eventual loss of cholinergic innervation not only contributes to the electrophysiological anomalies that affect the basal ganglia of PD patients, but also leads to the overactivity of the PPN’s resident glutamatergic neurons [17], which innervate dopaminergic (DAergic) neurons of the substantia nigra pars compacta (SNpc). It has been postulated that this effect could accelerate the DAergic degeneration seen in PD-affected brains [18]. Potentially, PPN-DBS could normalize basal ganglia electrophysiology and protect the remaining SNpc DAergic neurons [19, 20]. However, because of the PPN’s mixed neuronal population, it has, until the recent development of improved cell-type–specific modulatory tools, been impossible to hypothesize which neuronal subtype(s) deliver the clinical benefits associated with PPN-DBS.

In an earlier study, we utilized selective viral vector-driven expression of an excitatory Gq-coupled DREADD (designer receptors exclusively activated by designer drugs) in PPN cholinergic neurons of a unilateral lactacystin rat model of PD. In this animal model, selective stimulation of the remaining PPN cholinergic neurons by the DREADD agonist, clozapine *N*-oxide (CNO), for mimicking selective PPN cholinergic DBS, almost completely reversed the motor deficits seen in this PD model [11, 12]. This study supports the hypothesis that it is the cholinergic neuronal population, projecting from the PPN, which delivers some of the clinical benefits associated with PPN-DBS. Cholinergic axons project from the PPN to various basal ganglia targets, including the SNpc [21, 22], the thalamus [23, 24], and the striatum [25]; hence, the question remains as to which PPN cholinergic pathway is responsible for the clinical benefits associated with PPN-DBS. PPN cholinergic neurons projecting to the striatum, synapse with the nigrostriatal DAergic neurons, with studies in rodents, have demonstrated that stimulation of PPN cholinergic neurons leads to activation of nigrostriatal DAergic neurons [21, 26]. In the present study, we uniquely combined our PPN cholinergic DREADD-PD rat model with positron emission tomography (PET) brain scans, utilizing the DR2/3 ligand [^11^C]PHNO, to test our hypothesis that the CNO-induced motor improvement is due to PPN cholinergic-induced DA release from nigrostriatal DAergic terminals in the striatum. Additionally, we utilized the neuronal activity marker c-Fos to determine whether the CNO-driven motor benefits seen in this animal model of PD were due to changes in activity of neural circuits known to facilitate voluntary movement. In particular, we compared the activity between the prokinetic “direct” and inhibitory “indirect” pathway projection neurons that originate from distinct populations of striatal medium spiny neurons (MSNs) to project to different output structures. Finally, to ascertain whether PPN cholinergic projections to the thalamus contribute to the CNO-induced motor recovery seen in the current PD model, we measured c-Fos expression within neurons of the SNpc, as well as the ventrolateral (Vl) and ventromedial (Vm) thalamic subnuclei, with and without DREADD-based stimulation of PPN cholinergic neurons.

## Materials and Methods

### Laboratory Animals and Experimental Model

Animal experiments were approved by an ethics panel at Imperial College London (Ref: BMS39UNNGB2015) and were performed in accordance with the Animals (Scientific Procedures) Act, 1986 (UK) for the care and use of experimental animals as well as the European Communities Council Directive (2010/63/EEC).

We used choline acetyltransferase (ChAT)::Cre transgenic rats [27], in which Cre-recombinase is exclusively expressed within cholinergic neurons. Long–Evans hemizygous ChAT::Cre founder rats, obtained from the Missouri Mutant Mouse Regional Resource Centre (University of Missouri, USA) were bred with Long–Evans wild-type (WT) rats (Charles River Laboratories, Germany), for producing Cre^+^ offspring. Breeding of ChAT::Cre rats took place at the animal facility of Imperial College London, where the offspring underwent genotyping. For this, ear punches were collected from each rat at weaning age. Genomic DNA was extracted from each sample to genotype the *Cre-recombinase* transgene and therefore distinguish Cre-expressing from non-Cre-expressing littermate rats. Genotyping was performed by polymerase chain reaction, using the following primer sequences: 5″-AGCGATGGATTTCCGTCTCT (forward) and 5″-CACCAGCTTGCATGATCTCC (reverse). A positive band consisted of approximately 200 base pairs.

**Fig. 1:**
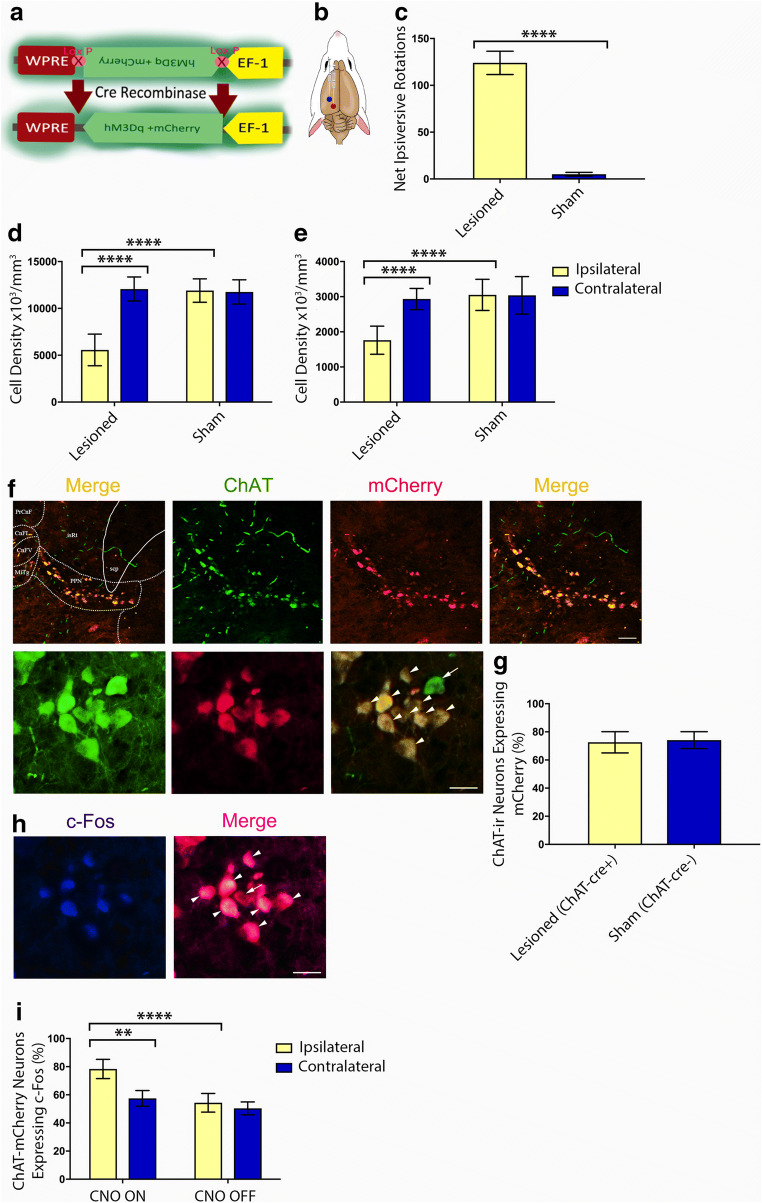
DREADD expression within unilateral rat PPNs. (A) DREADD incorporation into the host and its subsequent translation depends on Cre-recombinase, expressed under a ChAT promoter in ChAT::Cre transgenic rodents. In such animals, only cholinergic neurons possess the cellular machinery to express DREADD. (B) Stereotaxic injection of DREADD via an AAV vector into the left PPN (red dot). A second stereotaxic injection delivered lactacystin to the left SNpc (blue dot), rendering rats hemiparkinsonian. (C) The DAergic lesion produced amphetamine-induced ipsiversive rotations; sham controls demonstrated symmetric net rotations. (D) DAergic lesions were quantified by stereologically counting ipsilateral *versus* contralateral SNpc TH-ir neurons. (E) Stereological counts of the same rats revealed significant (48.5%) loss of ipsilateral PPN ChAT-ir neurons in lesioned rodents (*n* = 24) *versus* counts made in the contralateral hemisphere. Bilateral PPNs of sham control rats (*n* = 20) were left intact. (F) mCherry-tagged hM3Dq expressed robustly within Cre + PPN cholinergic neurons. The low magnification (×10 air) photomicrographs (top panel) show ChAT-mCherry-ir neurons stained in a representative sham-lesioned rat. The image on the far left shows the unmagnified version of the merged panel (on the far right in the same row of images), the latter having been cropped and zoomed in to accentuate the ChAT-mCherry-ir neurons’ confinement to the PPN. The mapped diagram delineates the PPN’s characteristic wedge-like shape, as defined by the nucleus’ resident cholinergic neurons and indicates the rat PPN’s anatomical location in relation to surrounding neural structures. The coronal brain section corresponds to Plate #99 (bregma − 7.92 mm) of a stereotaxic atlas of the rat brain [28]. Labeled areas correspond to the following brain regions: cuneiform nucleus, intermediate part (CnFI); cuneiform nucleus, ventral part (CnFV); microcellular tegmental nucleus (MiTg); precuneiform area (PrCnF); superior cerebellar peduncle (scp); isthmic reticular formation (isRt). Scale bar = 30 μm. The high magnification (× 40 air) photomicrographs (bottom panel) show the same neurons. The digital overlay shows all (arrowheads) but 1 (arrow) of the neuronal group being mCherry-ir. Scale bar = 50 μm. (G) Stereological counts of ChAT-mCherry-ir PPN neurons were similar between lesioned (72.6 ± 2%, *n* = 12) and control rats (74.1 ± 3.1%, *n* = 12). (H) CNO-induced c-Fos expression (indicating neuronal activation) within ChAT-mCherry-ir PPN neurons (arrowheads). The arrow shows a neuron *not* expressing c-Fos. Scale bar = 50 μm. The image in (F) (bottom panel) portrays the same ChAT-ir neurons shown in (H) (bottom panel). (I) Stereological quantification performed on toxin-lesioned animals of c-Fos expression within ChAT-mCherry-ir PPN neurons located on the hemispheric side ipsilateral to the lesion increased significantly (****p = 0.002) between CNO ON (*n* = 6) and CNO OFF (*n* = 6). Relatedly, during CNO ON, c-Fos expression levels within mCherry-ir PPN cholinergic neurons located ipsilateral to the lesioned hemisphere increased significantly compared with similar neurons on the contralateral PPN (**p = 0.0012). Histogram error bars depict SEM throughout

To test the present hypothesis, we stimulated PPN cholinergic projection neurons by overexpressing an excitatory DREADD (hM3Dq) consisting of a modified human M3 muscarinic receptor (hM3) fused to a fluorescent marker (mCherry) (Fig. 1A), which is selectively activated by the ligand, CNO [12, 29], but has no biological effect on native receptors. A Cre-dependent adeno-associated virus, serotype 2 (AAV2), delivered the hM3Dq construct stereotaxically into the left PPN, to facilitate DREADD expression within PPN cholinergic neurons of transgenic rats (Fig. 1B). The approach permitted restricted expression of the DNA encoding hM3Dq in rats’ cholinergic neuronal population, with spatial restriction to the PPN.

In our previous studies [10–13], we demonstrated that the unilateral nigral lactacystin model of PD not only produced a partial lesion of the DAergic nigrostriatal pathway, but also cholinergic neuronal loss in the PPN, thereby mirroring what is observed in PD patients, and hence highlighting the suitability of this model for the current study. Intracranial injections of ChAT::Cre rats with hM3Dq DREADD were carried out while the animal remained anesthetized for the lesion surgery (Fig. 1A). In total, 44 adult male rats (8-12 weeks old, weighing 300-400 g) were randomly assigned to 1 of 2 treatment groups: those receiving a sham (lactacystin vehicle only) lesion of the SNpc in combination with stereotaxic infusion of the DREADD-containing AAV2 into the PPN (*n* = 20) *versus* parkinsonian rats produced through intranigral, unihemispheric stereotaxic injection of the ubiquitin proteasomal inhibitor lactacystin [11, 12], combined with an intra-PPN infusion of the same excitatory DREADD-containing viral vector as used for the control animals (*n* = 24).

All rats underwent behavioral assessment at 2 time points: at baseline, conducted presurgically, when they had attained a weight of 250 g, whereas assessment was repeated at 5 weeks after surgery. During the later assessment, behaviors were conducted twice—in the CNO OFF state (following a vehicle-only intraperitoneal (i.p.) injection) and CNO ON state (following a 1-mg/kg i.p. injection of CNO in 0.9% normal saline with 10% DMSO (dimethyl sulfoxide)). Both lesioned (*n* = 6) and sham-lesioned rats (*n* = 4) were subjected to micro-PET imaging, in which the level of endogenous striatal DA release was quantified *in vivo* as the displacement of [^11^C]PHNO ligand from D2/3Rs. Subsequently, during CNO-mediated DREADD-based stimulation (CNO ON), the animals were sacrificed for gamma counter assessment of [^11^C]PHNO levels in the brain extracts. Stereological evaluation for validating the DAergic SNpc and cholinergic PPN lesions was carried out on all animals. Additional animals (lactacystin-lesioned: *n* = 6; sham-lesioned: *n* = 4) that did not undergo micro-PET scanning represented the baseline, non-DREADD stimulated (CNO OFF) state for gamma count measurements. A second group of lesioned (*n* = 12) and sham-lesioned rats (*n* = 12) were sacrificed (followed by transcardial perfusion to optimally preserve brain tissue for histological analyses), after they had completed the final behavioral test. Brain tissues from these animals were used for quantifying PPN cholinergic c-Fos-immunoreactive (ir), hM3Dq-mCherry expression, and striatal MSN D1/2R activation levels. For this set of animals, half the toxin-lesioned (*n* = 6) and half the sham-lesioned rats (*n* = 6) were sacrificed during CNO ON, whereas the rest of the cohort was sacrificed during CNO OFF (lactacystin-lesioned, *n* = 6; sham-lesioned, *n* = 6).

### Drugs

Guettier and others [30] showed that in mice that had received a single intraperitoneal injection of CNO (1 mg/kg), CNO plasma levels peaked after 15 min and only decreased 2 h after injections. This study, as well as the study by Alexander and others [31], reported that CNO remains systemically active in rodents for up to 10 h after administration. Such published data on CNO’s duration of action provided us with guidance as to the appropriate time window in which to carry out the CNO-induced behavioral and micro-PET assays. CNO (Enzo Life Sciences, Plymouth Meeting, PA, USA) was dissolved in DMSO and then diluted in a 0.9% sterile saline solution, to yield a final DMSO concentration of 10%. The drug solution was administered intraperitoneally at a dose of 1 mg/kg. CNO vehicle injections consisted of sterile saline containing 10% DMSO. D-amphetamine sulfate was purchased from Sigma-Aldrich and was administered at a dose of 5 mg/kg, dissolved in 0.9% saline. All drugs were injected intraperitoneally.

### Virus Production and Stereotaxic Injections

For generating the activating DREADD hSyn-DIO-hM3Dq-mCherry (containing the H134R mutation), we followed protocols reported in our previous work [12]. Briefly, the hM3Dq coding sequence [31, 32] fused in-frame with the fluorescent protein (mCherry) at the C terminus and driven by the elongation factor 1 alpha promoter was provided by the laboratory of Prof. Brian Roth (Viral Vector Core Facility, UNC, Chapel Hill, USA; Addgene plasmid ID: 44361). Our laboratory cloned the gene sequence in a Cre-dependent configuration into an AAV, to incorporate the gene of interest in a double-inverted open reading frame. Hence, when *Cre* is absent in a cell, the fusion construct hM3Dq-mCherry is retained in an inverse, nonsense orientation, thus limiting DREADD expression to ChAT-ir neurons in these ChAT::Cre rats. A flip-excision switch was added to allow stable transgene inversion [33]. The AAV viral construct was purified through a series of sucrose and cesium chloride ultracentifugation steps and dialysis, sequence verified, and then packaged in AAV2 serotype coat proteins by Vector Biolabs (Philadelphia, USA). Final pellets were suspended in 0.1 M phosphate-buffered saline (PBS) at a titer of approximately 1.4 × 10^13^ genomic copies/ml. Aliquots of virus lots were stored at − 80 °C until needed for stereotaxic injection.

To selectively express the hM3Dq receptors in cholinergic (ChAT-ir) neurons in the unilateral (left-sided) PPN of the rats, we injected the AAV2 vector containing the fused hM3Dq construct through a 2-mm burr hole drilled over the PPN, using the coordinates: anterior–posterior (AP) − 7.8 mm and medio-lateral (ML) + 1.8 mm (relative to bregma) [12]. The injection was made using a removable 32-gauge needle (7762-05, Hamilton, Reno, USA), attached to a 10-μl injector syringe (700 series, Hamilton) and driven by a microinjector syringe pump (11 Plus, Elite Series, Harvard Apparatus, Holliston, USA). The construct (1.5 μl, delivered at a constant rate (0.2 μl/min)) was delivered at a depth of − 7.0 mm (ventral–dural) relative to the dural surface of the brain. The needle was then lowered further 0.2 mm, to reach a depth of − 7.2 mm, where a further 1.5 μl was delivered, at the same rate as the first viral delivery. For delivering 1 μl lactacystin solution (4 μl of 2.5 μg/μl, Enzo Life Sciences, UK) or vehicle (sterile saline, pH 7.4), a second bore hole was drilled through the cranium over the SNpc, using coordinates we reported in our previous work [10–13]. Sham- and toxin-lesioned groups were operated on in a randomized manner, during the same surgical session.

Following the injections, the needle was left *in situ* for 5 min, before slowly retracting it from the brain. The skin incision was then closed with 4-0 monofilament nylon suture (Ethicon, Somerville, USA), with rats then left to recover in a heated recovery chamber. Fluid replacement (5 ml glucosaline, containing 0.18% NaCl and 4% glucose; Baxter Health Science Ltd., UK) was administered intraperitoneally after surgery. Rats were assessed daily for weight, general appearance, and the sutures’ condition for at least 7 days following surgery. At 7 to 10 days after surgery, the sutures were removed.

### Behavioral Testing

All rats (*n* = 44 in total; lactacystin-lesioned: *n* = 24; sham-lesioned: *n* = 20) utilized in this study, regardless of the experimental arm they were assigned to (to either incorporate micro-PET imaging or not), were subjected to the full battery of behavioral tests. All toxin- and sham-lesioned rats were subjected to behavioral testing before and after CNO-induced neuronal activation, with data analysis that was performed by an investigator blind to the experimental grouping of the animal test subjects. The first assessment was performed at baseline (prior to surgery), once rats had reached a minimum weight of 250 g, whereas the second was performed at 5 weeks after surgery. For both the first and second testing sessions, all rats were tested on the same day for the full test battery, with testing for CNO ON that commenced at 40 min after administering CNO. Amphetamine-induced rotations were performed on a single occasion, at 3 weeks after surgery to confirm lesioning status, by counting the number of clockwise and anti-clockwise rotations a rat made over a 15-min period, commencing 20 min after amphetamine dosing.

### Open Field

The rats’ activities when placed in a white plexiglass open field (OF) arena (90 cm long × 90 cm wide × 60 cm deep) were recorded (5 min) by a video camera suspended above the OF. Each animal’s movements were tracked digitally using ANY-Maze software (Stoelting ANY**-**Maze, Wood Dale, USA), supplemented by keystroke inputs of specifically observed behaviors, namely rearing and grooming. A total of 20 parameters were assessed, covering a range of motor functions: total distance traveled (m), average speed (m/s), maximum speed (m/s), time spent mobile (s), time spent immobile (s), number of ambulations, duration of ambulations (s), average number of ambulations, frequency of ambulations, number of immobile bouts, total number of rotations, total number of clockwise rotations, total number of anti-clockwise rotations, number of rearing episodes, duration of rearing (s), rearing frequency, number of grooming episodes, duration of grooming bouts (s), and grooming frequency.

### Limb-Use Asymmetry (“Cylinder”) Test

Rats were subjected to the limb-use asymmetry (“cylinder”) test, for assessing the animals’ use of the ipsilateral (unimpaired), contralateral (impaired), and both limbs making contact with the inner wall of a transparent plexiglass cylindrical enclosure (diameter 20 cm; height 40 cm), also referred to as “wall placement.” Rats were also assessed for the degree of inner wall exploration undertaken with the forelimbs, termed “serial-stepping” [11, 12]. Placement of a rat inside the cylinder encouraged upright support against the cylinder’s walls [34, 35], revealing forelimb asymmetries and allowing for comparing usage by the affected limb to that by the unaffected limb, because of the unilateral administration of lactacystin that produced motor asymmetry. For all testing phases (baseline/CNO OFF and CNO ON), 10 rears/rat were video recorded. A rat’s movements were scored by means of slow-motion playback of the recording. Instances were recorded where a rat made sole use of the ipsilateral (to the lesion) or contralateral forelimb or the simultaneous bilateral use of both forelimbs for upright support. An asymmetry score was calculated using the formula: % contralateral forelimb use = (number of contralateral forelimb placements + ½ number of bilateral forelimb placements/number of contralateral + ipsilateral + bilateral forelimb placements) × 100 [35].

### Postural Instability Test

The postural instability test (PIT) was performed as previously described [11, 12, 35]. In brief, a rat was held at 45° in a “wheelbarrow”-like position over a sandpaper-covered surface, with the tip of the rat’s nose being aligned with the 0 line of a ruler. The experimenter restrained 1 forelimb against the animal’s torso while moving the animal forward over the planted forelimb until it made a “catch-up” step to regain its center of gravity. The new position of the tip of the nose was then recorded. An average of 3 trials per forelimb per testing session represented each rat’s respective final score.

### Vibrissae-Evoked Forelimb Placement Test

We made use of the vibrissae-evoked forelimb placement (VEFP) test, which assesses sensorimotor integration across the midline [35]. Briefly, the animal was held by the torso with its forelimbs hanging freely. The animal was moved slowly to the edge of the countertop, until the vibrissae of 1 side brushed against the edge of a countertop. Intact animals typically place their forelimb rapidly onto the table surface, in response to ipsilateral vibrissae stimulation. In contrast, rats with unilateral lesions present with deficits in placing the limb contralateral to the lesion. Animals were assessed on 10 trials per side per test session. In contrast, rats with unilateral lesions would present with impairments in the placing response of the limb contralateral to the lesion. Placing asymmetries were presented as the percentage contralateral and ipsilateral forelimb placements per trial.

### Accelerating Rotarod Task

Motor coordination testing was performed on an accelerating SDI Rotor-Rod System (San Diego Instruments Inc., San Diego, CA, USA), with the use of an accelerating rather than constant speed protocol that was shown to minimize interference from learned memory [36]. An initial speed of 5 rotations/min (rpm) was systematically increased to 1 rpm/10 s. Latency to first fall (s) was recorded on 3 occasions, with an arbitrary upper limit of 500 s. To limit the effect of motor learning, animals were subject to no more than 3 trials. Trials were performed at baseline and repeated at 5 weeks after surgery, in the latter case with vehicle-only (CNO OFF), followed by a CNO intraperitoneal injection (CNO ON).

### [^11^C]PHNO Radiolabeling

PHNO (4-propyl-3,4,4a,5,6,10b-hexahydro-2H-naphtho[1,2-b][1,4]oxazin-9-ol) is a DA agonist with predictable DR2/3 sensitivity [37, 38]. When radiolabeled with ^11^C (or tritiated), it has been shown to have functional use in assessing receptor availability in both experimental animals and humans [39]. We utilized [^11^C]PHNO to determine the effect of CNO administration on endogenous DR binding in the striata of rats expressing the hM3Dq DREADD receptor in the PPN. The radiosynthesis of [^11^C]PHNO was performed as described elsewhere [37]. In brief, for the synthesis of radiochemically pure [^11^C]PHNO as a sterile, pyrogen-free solution, [^11^C]-amide was generated by reacting [^11^C]-propionyl chloride with 9-hydroxynaphthoxazine. Subsequently, this was reduced by lithium aluminum hydride and then purified with high-performance liquid chromatography.

### Micro-PET Imaging

hM3Dq-expressing rats (lactacystin-lesioned, *n* = 6; sham-lesioned, *n* = 4) were anesthetized by isoflurane inhalation, with respiration, pulse rate, and temperature monitoring (kept constant at 37 °C via a heating mat and/or heating lamp). Rats were placed into the bore (12 cm) of an Inveon Micro-PET scanner (Siemens, Germany). Each rodent was subjected to 2 scans. The first was during CNO OFF (at 40 min after receiving CNO vehicle (saline containing 10% DMSO, 1 mg/kg i.p.)). Scanned animals were recovered for a minimum of 24 h after the DMSO vehicle scan, before a second CNO ON (1 mg/kg, i.p.) scan was performed at 40 min after administering CNO. For administering the radioligand (5.2 ± 0.4 MBq [^11^C]PHNO), a direct tail vein cannulation (25 G angiocath) was performed under a topical EMLA® cream anesthetic. An attenuation correction CT scan was performed (lasting 12 min in total) prior to a 60-min dynamic PET scan. The data was histogrammed and decay corrected to the injection time. The images were then reconstructed using 2D filter back projection. Immediately following completion of the scan imaging protocol (constituting CNO ON), rats were sacrificed, and their brains removed. For a direct tissue comparison of radioligand uptake in the brain tissue of the scanned CNO ON group of rats, a CNO OFF group of rats was included for measuring emitted gamma radiation by gamma counter measurements. For this, additional hM3Dq-expressing rats (lactacystin-lesioned: *n* = 6; sham-lesioned: CNO OFF *n* = 4) underwent a radioligand biodistribution study. Outside of the scanner, these rats were anesthetized, their physiological functions monitored, given a CNO vehicle injection (i.p.) as described above, and then administered the radiotracer via direct tail vein injection. The radiotracer was allowed to distribute in these animals for the same length of time as was allowed for the scanned animals (60 min). Following animal sacrifice, the brains of both groups of rats that underwent micro-PET imaging (CNO ON) and the additional nonscanned ones (CNO OFF) were removed, with cerebellums and striata that were dissected bilaterally. The remainder of the brains were kept for subsequent stereological evaluation of the SNpc and PPN brain regions.

### Micro-PET-Derived Image Analyses

The regions of interest, the striata and cerebellums, were drawn by an independent observer blinded to the experimental grouping of the animal test subjects, by using the co-registered CT-PET images. To generate the *in vivo* binding potential (BP_ND_) values, custom Matlab software (MathWorks, Natick, MA, USA) was used to apply a simplified reference tissue model (SRTM) using the cerebellum as a tissue reference region. ∆BP_ND_ denotes the change in DAergic receptor binding (pre-CNO scan − post-CNO scan). For tissue activity measurements derived from the gamma counter measures, individual data were decay corrected to the time of injection, with data that was expressed as standardized uptake values ((SUV) = %injected dose per gram/weight of animal (kg)). The data was then normalized to cerebellar activity (representing a minimal DR2/3 binding reference region). Data are expressed as SUV (ratio to cerebellum) group means ± standard error of the mean (SEM).

### Animal Sacrifice and Tissue Processing

After animals subjected to either PET imaging or cut-and-count analyses were humanely sacrificed, decapitated, and their brains extracted, the cerebellums and striata were dissected out, with the remaining tissue (containing the SNpc and PPN) that was either snap-frozen in dry ice prechilled isopentane or postfixed in 4% paraformaldehyde (PFA) prior to immunostaining. After fixing in 4% PFA for 24 h, the tissue was placed in 30% sucrose solution for cryoprotection. Cryoprotected brains were stored at − 80 °C prior to sectioning. For analyzing the brains of rats that had not received a PET scan/cut-and-count evaluation, the rats were sacrificed by sodium pentobarbital overdose and transcardially perfused with 50 ml heparinized PBS (37 °C), followed by 4% PFA. The extracted whole brain was kept in 4% PFA for 8 to 12 h, then cryoprotected using a 30% sucrose solution before freezing for storage at − 80 °C. The brains containing the regions of interest (ROIs), namely the striatum, SNpc, PPN, and Vl and Vm thalamus, were coronally sectioned (30 μm) with a cryostat (Bright Instruments, UK) with sections mounted serially (from most rostral to most caudal) onto glass slides (VWR International, UK).

### Immunohistochemistry and Immunofluorescence

Immunohistochemistry (IHC) staining of the coronal brain tissue sections containing the PPN was performed to detect the rate-limiting enzyme ChAT, needed for acetylcholine (ACh) synthesis. After removing the sections from the − 80 °C freezer, they were left at room temperature (RT) to dry. Sections were then circled with a hydrophobic slide marker pen (PAP Pen, Zymed, San Francisco, CA), before hydrating through a graded series of ethanol (EtOH, Sigma) and then placing under running tap water for 5 to 10 min. The sections were incubated for 30 min in 0.3% H_2_O_2_ (Sigma) made in 0.05 M Tris buffered saline (TBS) before applying the primary antibody solution (1:300, polyclonal goat, AB144P, Millipore, MA, USA) overnight at RT. The next morning, excess primary antibody was washed off (3 × 3 min) with 1% PBS followed by incubation in 20% horse serum (Vector Laboratories, UK) for 1 h, before applying the biotinylated secondary antibody (1:100, horse anti-goat, BA-9500, Vector Laboratories) to the sections for 2 h at RT. Again, excess secondary antibody was washed off with PBS before a third-layer avidin–biotin–peroxidase complex (ABC Elite, Vector Laboratories) was applied to the sections for 1 h at RT. Visualization was performed using 3,3′-diaminobenzidine (Vector Laboratories) with cresyl fast violet counterstaining applied for 3 to 5 min. Sections were dehydrated in graded EtOH, cleared with xylene, and finally mounted using DPX (Sigma). The slides were left to dry completely before microscopic analysis. For IHC staining of tyrosine hydroxylase (TH)-ir neurons in SNpc-containing coronal rat brain sections, a similar procedure was followed using an anti-TH primary antibody (1:300, polyclonal rabbit, P40101-0, Pel-freeze, AR, USA). After blocking for nonspecific binding with 20% horse serum, horse anti-rabbit biotinylated secondary antibody was applied (1:100, Vector Laboratories) for 1 h at RT.

Because the transcription factor c-Fos serves as an indirect marker of neuronal activity, which upregulates in response to increased neuronal activity [40], we used a c-Fos confocal immunoassay to quantify the degree of cholinergic PPN neurons resulting from CNO-DREADD excitation. c-Fos immunostaining was performed as described previously [12], using a sheep polyclonal primary antibody (1:500, AB1584, Merck Millipore). Animals were deeply anesthetized with sodium pentobarbital (50 mg/kg) 40 min after administering CNO (i.p.) and then transcardially perfused, and the brains were removed, postfixed, and then cryoprotected, as described above. Similarly, dual immunofluorescent staining of thalamic neurons comprised of an anti-NeuN primary antibody (1:700, rabbit polyclonal, ABN78, Merck Millipore) with an anti-c-Fos one (detailed above), fluorescently tagged with Alexa-Fluor® 488 and 546 secondary antibodies (both at 1:200, Thermo Fisher Scientific), respectively.

To determine the level of hM3Dq-mCherry expression within PPN cholinergic neurons, on the hemispheric side ipsilateral to the DREADD delivery site, sequential slides containing the sectioned PPN were identified by gross anatomical comparison against a stereotaxic atlas [28]. Every sixth section was stained with an anti-ChAT antibody and fluorescently tagged with a secondary antibody excitable at 488 nm, as described above. The DREADD construct carries an mCherry tag which excites maximally at 587 nm.

Because the activity of DR1-bearing neurons could not be assessed from the results of the [^11^C]PHNO binding assay, as this radioactive agonist rather has DR2/3 selectivity [38], triple fluorescent staining was performed on striatum-containing brain tissue sections to determine relative levels of striatal MSN activation, mediated by PPN cholinergic neuronal activation. The sequential sections containing striatal tissue were identified by gross anatomical comparison with a stereotaxic atlas [28]. The sections were stained with c-Fos (detailed above) to quantify neural activity in DR1 (1:300, sc-33660, monoclonal mouse, Santa Cruz, every 6th section) and DR2-bearing (1:300, sc-5303, monoclonal mouse, Santa Cruz) striatal MSNs (also using every 6th section in the series), comparing sections from rats taken before and during PPN cholinergic stimulation. Simultaneously, sections were stained with an antibody raised against DARPP-32 (1:300, sc-11365, polyclonal rabbit, Santa Cruz), to identify striatal MSNs. The blocking solution consisted of 20% donkey serum, applied to sections for 1 h at RT. Antibody-binding sites were then visualized using Alexa-Fluor® 546 (donkey anti-sheep) for tagging c-Fos, Alexa-Fluor® 488 (donkey anti-mouse) for identifying DR1 and DR2, and/or Alexa-Fluor® 405 (donkey anti-rabbit) for DARPP-32. All secondary antibodies were purchased from Thermo Fisher Scientific and each was applied at a dilution of 1:200. All antibodies and sera were diluted in TBS containing 0.1% Triton X-100 (Sigma).

For optimizing the immunofluorescence staining protocols applied here, we consulted published protocols that utilized the same primary antibodies applied to rodent brain tissue sections. This included for detecting DR1 and DR2 [41–43]. For testing each primary antibody staining assay, a “procedural control” stain was included, in which the primary antibody was omitted, but otherwise the same procedure was used, including incubating the samples with the relevant secondary antibody. In these brain tissue sections, no immunolabeling was observed, to affirm that the secondary antibody did not in any of the reactions bind nonspecifically to certain cellular components. In addition, we consulted published guidelines to determine alternative brain regions known to express the various target antigens, to thereby comprise positive tissue control reactions, which were included in the optimization and quality control steps. For example, for immunolabeling DR1 and DR2, we concomitantly stained brain tissue sections containing the medial frontal cortex, as per previously reported experiments [44]. On each occasion, such positive control specimens emitted intense fluorescence signaling.

### Stereological Neuronal Counts

Stereological cell count estimations were performed using the optical fractionator technique [45]. Stained brain sections containing the ROIs (SNpc and PPN) were digitally scanned with a Nikon Eclipse E800 microscope, equipped with a 3CCD camera (JVC Ltd., London, UK) under a low magnification objective lens (× 2.5 air-immersion). On the scanned tiled images, the ROIs were manually delineated using Image-Pro Plus image analysis processing software (v. 9.1, Media Cybernetics, Inc., Bethesda, MD, USA), guided by anatomical landmarks [28]. Counting frames (SNpc 160 × 140 μm, PPN 150 × 150 μm) were generated within the respective ROI, with neurons of interest that were counted via manual input at × 10 (air) magnification by a single investigator blinded to the animal treatment. The height sampling fraction (hsf) was calculated as the height of the optical dissector, measured by using a Heidenhain microcator (Hedenhain, Germany), relative to the actual section thickness (30 μm). The section sampling fraction (ssf) was set at 1/6, because every 6th section was included in the analysis. Total cell estimates were then obtained by using the formula: *N* = *n*(1/ssf)(1/asf)(1/hsf), where *n* equals the number of positive cells counted and the area sampling fraction (asf) represents the total area of the counting frame, relative to the ROI area, as previously described [45].

The degree to which PPN cholinergic neurons contained hM3Dq-mCherry expression was quantified by performing 2-channel fluorescent scanning using a Nikon Eclipse E8 microscope with motorized stage (Nikon Instruments, UK). Image tiling was performed using Surveyor software, to create 2-channel overlays. Separate counts were made for ChAT-ir and ChAT-ir/mCherry-ir co-expressing neurons. Sequential sections containing the SNpc and PPN were separately immunofluorescently (double)-stained with either c-Fos-TH or c-Fos-ChAT. c-Fos expression was quantified in SNpc DAergic neurons and PPN cholinergic neurons using stereology, for rats sacrificed during CNO ON *versus* CNO OFF state, and in both cerebral hemispheres. Triple-stained c-Fos-DR1-DARPP-32/c-Fos-DR2-DARPP-32 sections were tiled and quantified using the same method and stereological parameters as for PPN lesion quantification, generating instead counts for DR1- and DR2-bearing MSNs that expressed c-Fos *versus* those not expressing c-Fos on both lesioned *versus* unlesioned striatal sides and in CNO-DREADD stimulated *versus* nonstimulated rodents.

Similarly, for the thalamic neuronal counts, every 6th section was selected for staining, with slide scanning of stained slides and stereological quantification which occurred as described for the PPN lesion studies. Neuronal count estimations were generated for rats during CNO OFF and CNO ON and on lesioned *versus* nonlesioned cerebral sides. Thalamic subdivisions were identified based on stereological atlas anatomy [28], enabling cell counts to be generated for different thalamic subdivisions.

### Statistical Analyses

An online calculator (http://powerandsamplesize.com) was used for estimating statistical power and effective sample sizes. For computing such values, type I errors were controlled for by setting the significance level at 5%. Furthermore, a 2-tailed direction of effect was applied, which is standard in animal research [46]. For determining the sample size, a 2-sample Student’s *t* test was used for comparing the means obtained by sham control rats to lesioned animals.

Data is presented as mean ± SEM throughout. The *α* level was set at 0.05 for all analyses and performed using GraphPad Prism 7.0 (GraphPad Software, La Jolla, CA, USA). *p* values were designated as follows: ****p < 0.0001 and ***p < 0.001, extremely significant; **p ≤ 0.01, highly significant; *p ≤ 0.05, significant; and *p* > 0.05, n.s. All data points were examined on scatterplots to evaluate normality. For multiple comparisons, 1- or 2-way ANOVA tests were employed as appropriate, with Bonferroni post hoc corrections. For comparing the effects in the sham *versus* lesioned animals, a Student’s *t* test was used, whereas a Mann–Whitney test was used to specifically compare the effects of DREADD-based stimulation on SUV values in striatal tissue (normalized to the cerebellum) between the lesioned and nonlesioned animals.

## Results

### Stereological Cell Counts Confirm Lactacystin-Induced Nigral Dopaminergic and PPN Cholinergic Lesions

To validate the ability of an intranigral lactacystin infusion to produce a rat lesion model that mimics human PD neuropathology, a series of behavioral and IHC-based investigations were conducted. Amphetamine-induced contralateral rotations made by the rats were counted at 3 weeks after surgery, as an index of the lesion deficit [47]. Lesioning was considered successful if rotational behavior demonstrated at least 7 anti-clockwise (a.k.a. ipsiversive) rotations per minute, a threshold also used in our previously published work [11, 12]. Scores were significantly higher for the lesioned group, whereas the sham controls demonstrated essentially symmetric net rotations (****p < 0.0001) (Fig. 1C).

Nigral DAergic neuronal counts were quantified using stereology for each sham (*n* = 24) and lesioned animal (*n* = 24), in both the injected (left/ipsilateral) and nonlesioned (right/contralateral) cerebral hemispheres. At 5 weeks after surgery, lactacystin lesioning produced a significant 53.9% reduction in DAergic neuronal density on the lesioned side, producing a total neuronal count of 5565 ± 398 (mean ± SEM), compared with the nonlesioned side (12,075 ± 303) (****p < 0.0001) (Fig. 1D), assessed as TH-ir, the rate-limiting enzyme in DA synthesis. SNpc DAergic neuronal counts for control rats were similar between the sham-lesioned ipsi- (11,910 ± 511) and nonlesioned contralateral sides (11,762 ± 530) (*p >* 0.05, not significant (n.s.)) (Fig. 1D). Lesioned animals showed a 48.5% loss of ipsilateral PPN cholinergic neurons (1762 ± 400) compared with the contralateral PPN ones (2934 ± 302) (****p < 0.0001) (Fig. 1E), a significant reduction also compared with ipsilateral PPN cholinergic neurons of sham-lesioned controls (3052 ± 444) (****p < 0.0001) (Fig. 1E). Brain material was derived from rats that had either been transcardially perfused (lactacystin-lesioned: CNO ON *n* = 6, CNO OFF *n* = 6; sham-lesioned: CNO ON *n* = 6, CNO OFF *n* = 6) or where the brain tissue had been cryopreserved (lactacystin-lesioned: CNO ON *n* = 6, CNO OFF *n* = 6; sham-lesioned: CNO ON *n* = 6, CNO OFF *n* = 6), as the brain tissue was also required for measuring tissue-associated gamma radiation. Negligible-only measurement differences were seen between these 2 groups of rats that differed as to the method by which the brains were collected and preserved for postmortem analysis, for either SNpc DAergic or PPN cholinergic neuronal counts. This provided assurance to allow us to pool data for all lesioned and all sham control rats for this analysis.

### Viral-Mediated hM3Dq Expression

In lactacystin-lesioned rats, stereological counts made of PPN (lesioned hemisphere) cholinergic neurons (identified by fluorescent (green) tagging of ChAT demonstrated robust expression of hM3Dq-mCherry (Fig. 1F), with a 72.6 ± 2% of all PPN cholinergic neurons that showed overlap (Fig. 1G). This was similar between both hemispheres of the sham controls, which showed a 74.1 ± 3.1% overlap (Fig. 1G). In all cases, representing both lesioned and sham control animals, no hM3Dq-mCherry expression, in the absence of ChAT co-expression, was observed in any of the stained sections. Taken together, these results indicate that our use of Cre-dependent viral vectors, to permit restricted expression of hM3Dq in neurons defined by the expression of the cholinergic genetic marker ChAT, is an effective strategy for targeting the PPN cholinergic neurons of rats, in order to enable cell-type-specific manipulations.

The ability of CNO as a muscarinic DREADD ligand, to activate cholinergic PPN neurons expressing hM3Dq, was verified by stereologically quantifying immunofluorescence of the immediate early-gene c-Fos, its expression correlating with neuronal activation in a well-described cellular pathway [48]. We co-analyzed the expression of c-Fos (blue) with neurons expressing the DREADD-mCherry fusion protein, to identify virally infected neurons in this brain region (Fig. 1H). The use of a Cre-dependent fluorescence reporter rat line allowed for restricting DREADD expression to cholinergic neurons, with stereotaxic microinjection of the virus allowing for spatial specificity of DREADD expression to the PPN. The number of mCherry-ir cholinergic neurons of the PPN that co-stained for c-Fos was stereologically quantified for both ipsilateral and contralateral hemispheres in the postmortem brains of lactacystin-lesioned rodents, by comparing the PPNs of CNO-unstimulated (CNO OFF, *n* = 6) with CNO-stimulated (CNO ON, *n* = 6) rats (Fig. 1H). During CNO OFF, the mean ipsilateral c-Fos expression in DREADD-transfected PPN neurons of lesioned animals was 50.4 ± 4.1% compared with 54.3 ± 3.4% contralaterally, giving a negligible interhemispheric difference of 3.9%. In contrast, during CNO-DREADD stimulation, this interhemispheric difference increased to 12.6 ± 1.3% because of disproportionate high ipsilateral c-Fos expression levels of PPN cholinergic neurons (78.4 ± 6.8%), compared with similar neurons on the contralateral side (54.3 ± 5.2%) (**p = 0.0012). Viewed alternatively, stereological counts made of c-Fos-expressing ChAT-mCherry-ir PPN neurons located ipsilaterally to the lesioned hemisphere increased significantly (****p = 0.002) from CNO OFF to CNO ON (Fig. 1I). Taken together, these results demonstrate robust DREADD expression within ipsilateral PPN cholinergic neurons, with stimulation via hM3Dq-CNO that resulted in significantly increased levels of cytoplasmic c-Fos protein, reflecting our previous findings [12].

### Stimulation of PPN Cholinergic Neurons in Parkinsonian Rats Reverses Motor Deficits

**Fig. 2:**
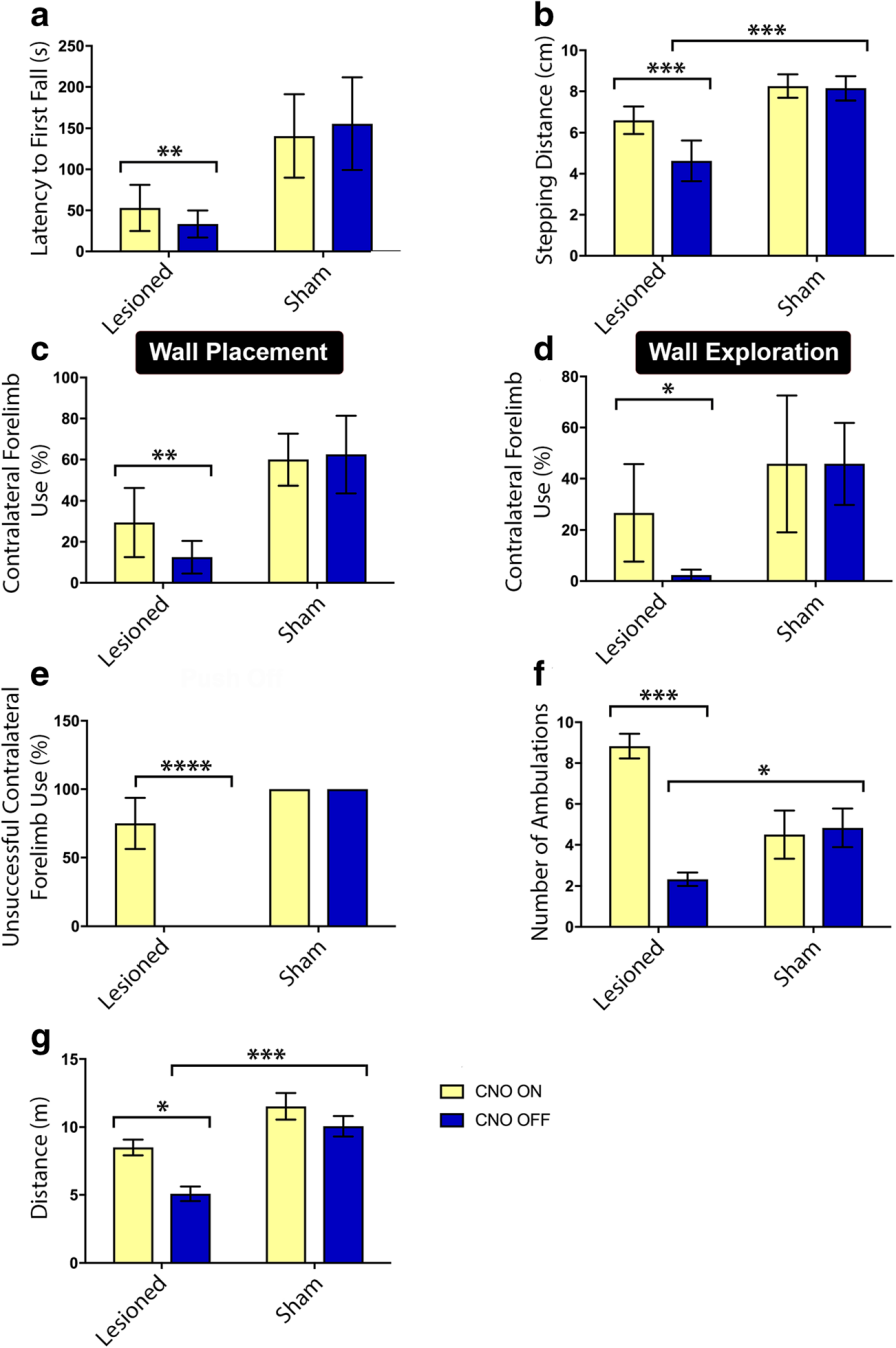
SNpc hemi-lesioning produced motor deficits across several domains, which recovered via DREADD-CNO stimulation of remaining PPN cholinergic neurons. (A) During accelerating rotarod testing of the lesioned rats, latency to first fall increased from 33.5 ± 16.5 s (CNO OFF) to 53 ± 28.2 s (CNO ON) (**p = 0.002). Sham-lesioned rodents displayed consistent, nonimpaired performance regardless of CNO-induced stimulation or not. (B) At CNO OFF, evaluation during the PIT revealed reduced average stepping distance because of PPN cholinergic and SNpc DAergic lesioning compared with sham control rats (***p < 0.001). Lesioned rats showed significant functional recovery during CNO ON (***p < 0.001). (C, D) The results of vertical cylinder “wall placement” and “wall exploration,” respectively. SNpc and PPN hemi-lesioning produced significant under-use of the contralateral forelimbs, with subsequent amelioration in performance during CNO ON. (C) **p = 0.007; (D) *p < 0.05). (E) For lesioned rats, dramatic loss of function at CNO OFF followed by recovery at CNO ON was particularly evident in the VEFP test, in which responses to vibrissae stimulation were terminated in all lesioned animals, but mean response rate recovered near-completely during CNO ON (****p < 0.0001). (F) In the OF arena, lesioned rats tended to freeze and/or display reduced motion, but recovered in this aspect during CNO ON (***p < 0.001). (G) For the same testing paradigm, the lesioned rats covered significantly less distance over the 10-min test period at CNO OFF compared with CNO ON (*p = 0.02) and also compared with sham control rats at CNO OFF (***p = 0.0003). Error bars depict SEM throughout. Lactacystin-lesioned: CNO ON *n* = 12, CNO OFF *n* = 12; sham-lesioned: CNO ON *n* = 10, CNO OFF *n* = 10

We previously showed that stimulation of DREADD-bearing cholinergic neurons of the PPN alleviates motor deficits across a number of domains. These findings were verified in the present study [12]. In the current study, we observed marked restoration of gait stability, locomotor asymmetry (measured via the vertical “cylinder” test), and sensorimotor integration across the cerebral hemispheric midline (assessed via the VEFP test) (Fig. 2), mirroring the putative effects of human PPN-DBS. The rats’ performance during accelerating rotarod testing provided an additional measure of postural instability beyond the parameters measured in our previous work [11, 12]. For this, latency to first fall markedly increased in the lactacystin-lesioned group when testing occurred during CNO ON (53 ± 28 s) compared with the CNO OFF phase of testing (33.5 ± 16.5 s) (**p = 0.002) (Fig. 2A). Sham-lesioned control rats showed consistent performance in this motor-related parameter, regardless of whether PPN cholinergic neurons were stimulated (*p >* 0.05, n.s.) (Fig. 2A).

The lesioned rats’ mean stepping distances, as was assessed in the PIT (Fig. 2B), were significantly impaired compared with control rats during CNO OFF (***p < 0.001). With DREADD-based stimulation, however, lesioned animals demonstrated near-perfect recovery in this parameter (***p < 0.001).

The rats also underwent the limb-use asymmetry “cylinder” test (Fig. 2C–F) measuring the rat equivalent of akinesia, tremor, postural deficits, and dyskinesia, which manifests when a rat has been placed inside a vertical cylinder and explores the inside by rearing and touching the walls of the cylinder, with its forelimb paws providing postural support [49]. Testing of lesioned rats during CNO OFF produced a significant performance reduction in several parameters, with subsequent recovery seen during CNO ON, including in “wall placement” (**p = 0.007) (Fig. 2C) and “wall exploration” (*p < 0.05) (Fig. 2D), but not for “push off” (*p >* 0.05, n.s. (data not shown)) nor landing (*p >* 0.05, n.s. (data not shown)). Control rats showed an overall consistent performance, with no significant changes seen between baseline measures and when assessment was repeated at 5 weeks after surgery.

Responses in the VEFP test (Fig. 2E) were significantly impaired in lesioned rats, but robustly present at baseline and in sham control rats. The response was almost entirely mute in lesioned rats during CNO OFF, but recovered near-perfectly during CNO ON, reaching near-baseline values (****p < 0.0001).

Finally, lesioned rats’ number of ambulatory bouts over 10 min in the OF (Fig. 2F) arena increased significantly during CNO ON compared with CNO OFF (***p < 0.001) as opposed to assessment that occurred at CNO OFF, in which compared with sham control rats, lesioned animals were significantly impaired in this measure (*p = 0.03). In addition, whereas lesioned rats covered significantly less distance in the OF arena than sham-lesioned rats over the testing period when CNO OFF testing was performed (***p = 0.0003), lesioned animals recovered in this parameter to a significant extent when this cohort’s performance at CNO ON was compared with CNO OFF (*p = 0.02) (Fig. 2G). All the other parameters remained unaffected (*p* > 0.05, n.s. (data not shown)). Sham-lesioned rats’ performance across all motor parameters assessed in the OF arena remained consistent, regardless of whether the CNO OFF or CNO ON stage was assessed. The lesioned rats’ performance in the locomotor parameter “number of ambulations” seems to have improved upon being administered CNO significantly more than any of the motor-related aspects assessed in our study, thereby deserving further attention in future work, particularly to better understand the mechanisms behind the seemingly large influence PPN cholinergic–driven stimulation exerts on this index of motor function. It seems unlikely this result reflects a tendency for PPN cholinergic stimulation to induce general hyperactivity in rats, as other indicators of locomotion in this test were not similarly affected by the same therapeutic intervention. It is known that OF arena size can significantly affect ambulations in rats and mice [50–52]. Future studies to determine whether the degree of behavioral rescue by DREADD-CNO could be altered by changing the dimensions of the OF arena will be insightful.

### [^11^C]PHNO PET and a Reduction of [^11^C]PHNO Binding Indicates Striatal DA Release in Consequence to DREADD-Based Stimulation of PPN Cholinergic Neurons

**Fig. 3:**
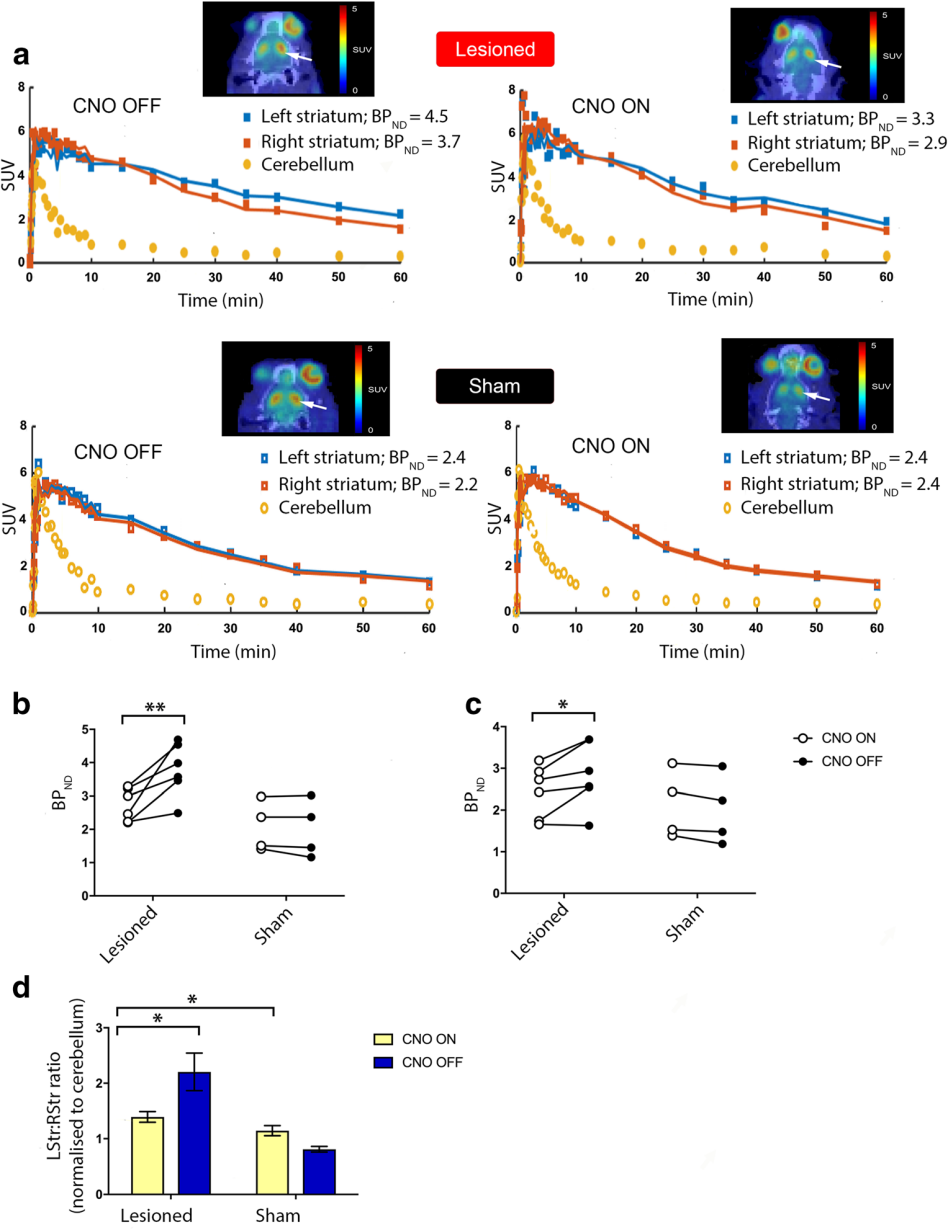
PET and BP_ND_ analysis indicate striatal DA release upon DREADD-based stimulation of PPN cholinergic neurons. (A) Representative PET/CT images at the level of the striatum, pre- (left) and post-CNO (right) in a representative lesioned (top row images) and sham-lesioned control rat (bottom row images). The toxin/sham-lesioned area is indicated by the white arrowhead. For each panel, the TACs of the right (contralesional) and left (ipsilesional) striata are reported, along with the SRTM BP_ND_ estimates obtained using the cerebellum as reference region. During CNO OFF, higher [^11^C]PHNO BP_ND_ values were found within left (ipsilesioned) striata compared with right (contralesioned) striata; receptor occupancy estimates were comparable at CNO ON. (B) BP_ND_ estimates in the ipsilesional and (C) contralesional striata for lesioned rats at CNO OFF (*n* = 6) and CNO ON (*n* = 6), as well as sham control rats (CNO OFF: *n* = 4; CNO ON: *n* = 4). In hemi-lesioned rats, BP_ND_ estimates were reduced in both the ipsilesional and contralesional sides during CNO ON compared with baseline; the reduction was particularly striking on the lesioned hemispheric side. No notable change in BP_ND_ estimates was seen between CNO OFF and CNO ON for sham rats. (D) Left-to-right [^11^C]PHNO striatal SUV ratios (normalized to the cerebellum) were significantly higher pre-CNO in lesioned *versus* sham rats (*p = 0.02). In lesioned rats, at CNO ON, there was a significant decrease in the L:R striatal SUV ratio compared with CNO OFF (*p = 0.017). Error bars depict SEM throughout

PET/CT imaging utilizing the DAergic agonist tracer [^11^C]PHNO allowed for noninvasive investigation of the functional effects of PPN-targeting cholinergic-specific stimulation on the nigrostriatal DAergic pathway in a well-validated rat model of PD. Four sham control and 6 lesioned animals underwent a scan at baseline (CNO OFF), followed by a second scan for CNO ON imaging. A minimum of 24 h was permitted between scans to allow for radiotracer washout and recovery from anesthesia. The [^11^C]PHNO BP_ND_ was then estimated by fitting a SRTM to the time–activity curves (TACs) that had been generated for the striata and cerebellums. The cerebellum served as a reference region, replacing a plasma input function to correct for intersubject differences in the pharmacokinetic delivery of the tracer to the brain tissue (Fig. 3A). For the hemi-lesioned rats, [^11^C]PHNO BP_ND_ during CNO OFF was consistently higher for the ipsilesioned striata (3.8 ± 0.3) compared with the intact striata (2.8 ± 0.3) (*p *<* 0.05). This effect may be due to a combination of factors, including a loss of endogenous DA release which would normally displace [^11^C]PHNO, or be due to reduced striatal DA levels, which will in itself trigger a compensatory increase in DR2-like expression, making target neurons more sensitive to the lower levels of DA.

In the ipsilesional striata of the parkinsonian rats, CNO-induced stimulation significantly reduced [^11^C]PHNO binding (∆change% 26 ± 5.8) (**p *<* 0.01) (Fig. 3B) compared with the CNO OFF phase, which is consistent with an increase in DA concentration. In the same animals, CNO ON produced a reduction in [^11^C]PHNO binding in the contralesional striata (∆change% 14 ± 6.2) (*p < 0.05) (Fig. 3C), but to a significantly lesser extent than was seen in the ipsilesional striata (Fig. 3B). In sham control rats, the BP_ND_ values taken at baseline (CNO OFF) showed no significant differences between the left striata (ipsilateral to the sham-injected SNpc) (2 ± 0.4) and right striata (on the intact hemispheric side) (1.99 ± 0.3) striata. CNO-induced stimulation did not significantly change the interhemispheric striatal BP_ND_ of sham control rats when compared with this cohort’s CNO OFF values (∆change% − 6.09 ± 5.3, n.s.) (Fig. 3B, C).

>To support the *in vivo* imaging findings, the levels of radioactivity associated with *ex vivo* tissue was evaluated by gamma counting. For this, striata lying ipsilateral and contralateral to the intranigral stereotaxic injection site and the cerebellums were excised and then weighed before the associated radioactivity was measured. SUVs were calculated and normalized to the reference region (the cerebellum), for which counts were determined. Injected radioactivity dose was measured and decay corrected, as detailed in the “Materials and Methods” section. Normalized SUVs were expressed as left:right ratios (L:R striatal SUV) to enable inter-rat comparisons because of the potential spread of DREADD in the brain tissue and to control for intersubject differences in physiology and neurochemistry.

At CNO OFF, the mean [^11^C]PHNO L:R striatal SUVs of hemi-lesioned rats was 2.2 ± 0.34, producing a significant difference compared with the mean L:R striatal SUV ratio of sham controls (0.81 ± 0.05) (*p *=* 0.02) (Fig. 3D). This implies greater binding of the radiotracer on the lesioned side, consistent with lower endogenous DA concentration, which could be considered a compensatory increase in DR2 expression. CNO-induced activation produced a significant decrease (*p *=* 0.017) in the L:R SUV (1.39 ± 0.10) of the lesioned rats compared with this group’s CNO OFF values (2.38 ± 0.36); a similar significant decrease was not observed in sham-lesioned rats (1.2 ± 0.09, *p* *>* 0.05, n.s.) (Fig. 3D). For the hemi-lesioned rats, baseline [^11^C]PHNO BP_ND_ was consistently higher for the ipsilesional striata compared with the contralesional striata, consistent with lower endogenous DA concentration, a compensatory increase in DR2-like expression, or a combination of both. Taken together, these results suggest strongly that DREADD-CNO for activating cholinergic-only neurons projecting from the PPN induced striatal DA release on the lesioned hemispheric side of lactacystin-lesioned rats.

### PPN Cholinergic Stimulation Induces Upregulated Neuronal Activity of SNpc DAergic Neurons

**Fig. 4:**
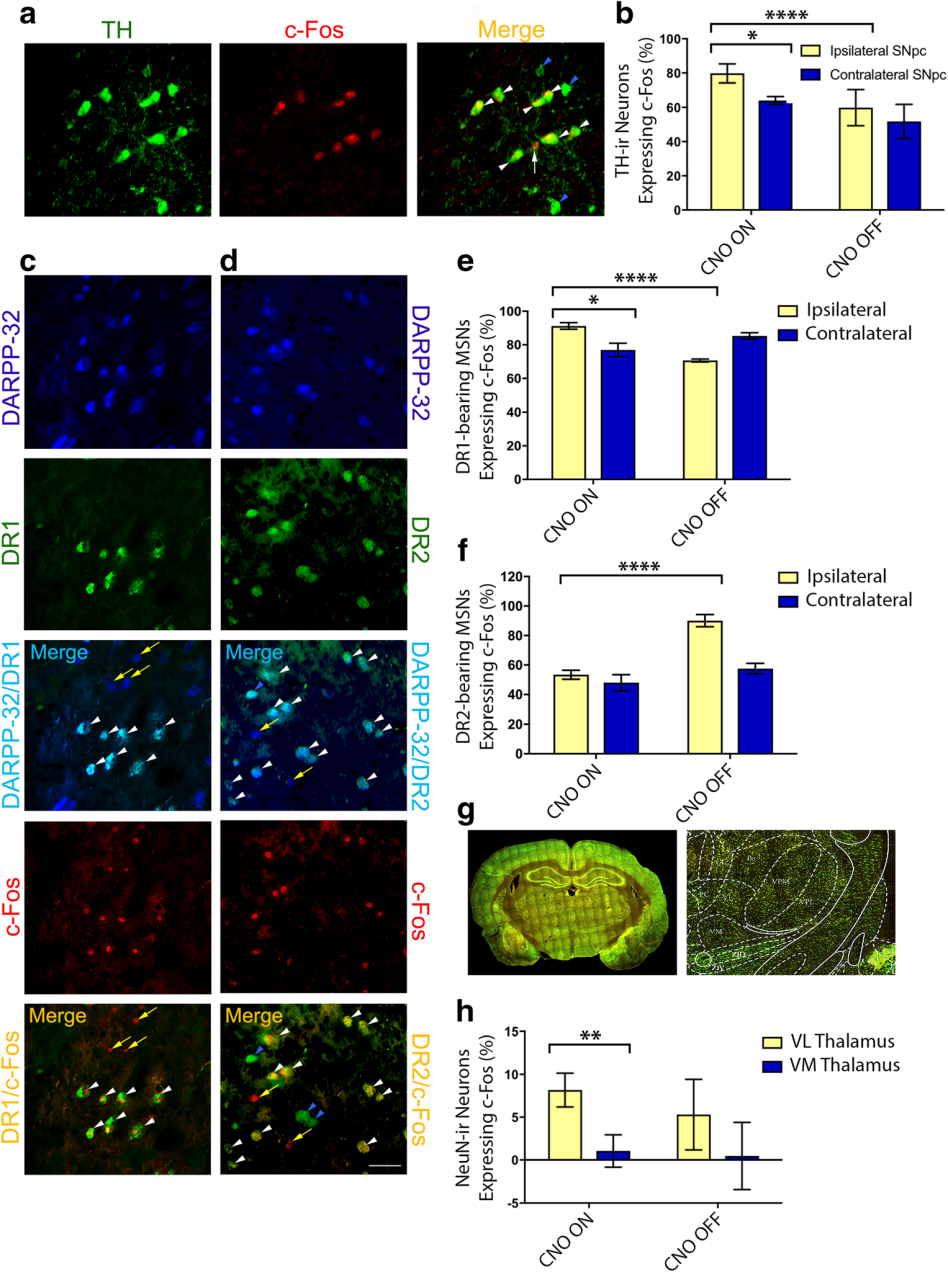
Activity effects of PPN cholinergic stimulation on striatal SNpc and thalamic neurons. (A) High-power photomicrographs of nigral TH-c-Fos co-stained neurons, indicating TH-c-Fos-ir neurons (white arrowheads), interspersed with c-Fos immunonegative TH-ir neurons (blue arrowheads) and a single non-DAergic c-Fos-ir neuron (arrow). Scale bar = 20 μm. (B) The lesioned hemispheric side (*vs* intact side) of parkinsonian rats displayed an overall reduction in the number of SNpc nigral TH-ir neurons expressing c-Fos. The mean c-Fos expression level of remaining SNpc TH-ir neurons on the ipsilateral (lesioned) side increased significantly (****p < 0.0001) from the CNO OFF (59.9 ± 10.6%, *n* = 6) to CNO ON state (79.8 ± 5.5%, *n* = 6). Also, during PPN cholinergic stimulation, SNpc DAergic neuronal activity increased substantially, revealed by an interhemispheric difference of 15.8% (*p = 0.04). (C) Single-channel and merged photomicrographs showing DARPP-32, DR1, and c-Fos-ir neurons, respectively. PPN cholinergic neuronal stimulation in lesioned compared with control rats increased the activity of striatal DR1-bearing MSNs. Scale bar = 50 μm. (D) Single-channel photomicrographs of DARPP-32, DR2, and c-Fos-ir neurons and 2 two-channel overlays showing DR2-MSNs and c-Fos-DR2 expressing MSNs. (C, D) D1/DR2-bearing c-Fos-ir MSNs (white arrowheads), DR2-bearing c-Fos immunonegative MSNs (blue arrowheads), and non-DR1/DR2-bearing c-Fos or MSNs (yellow arrows). Scale bar = 50 μm. (E) PPN cholinergic stimulation exerted an overall potentiating effect on ipsilateral striatal DR1-MSNs in lesioned (*n* = 6) compared with sham control rats (*p = 0.0182, *n* = 6). (F) In contrast, ipsilateral DR2-MSNs’ neural activity in lesioned rats compared with shams reduced significantly during CNO ON (***p < 0.0001) (*n* = 6). (G) A low-power photomicrograph (tiled image, left) reveals c-Fos immunofluorescence, at approximately coronal position − 2.3 mm (relative to bregma) of a rat brain stereotaxic atlas [28], with enlarged view shown on the right. Scale bar = 1 mm. (H) Compared with CNO OFF (*n* = 6), PPN cholinergic neuronal stimulation did not alter c-Fos expression levels within Vm and Vl thalami neurons, but substantially increased the expression in the Vl *versus* Vm (**p = 0.009, *n* = 6). Error bars depict SEM throughout

The number of bilateral SNpc DAergic neurons expressing c-Fos (Fig. 4A) was evaluated stereologically in brains collected from the culled toxin-lesioned rats. Analysis of the lesioned rats revealed that at CNO OFF, the difference in c-Fos expression between ipsilateral and contralateral hemispheres was negligible (*p >* 0.05, n.s.) (Fig. 4B). However, during CNO-induced stimulation of the PPN cholinergic neurons, the interhemispheric difference increased by 15.8% (*p *=* 0.04) (Fig. 4B). This change was due to a disproportionately high level of SNpc DAergic neurons expressing c-Fos ipsilaterally (79.8 ± 5.5%) during the activated state, compared with the same hemispheric side but prior to CNO administration (59.9 ± 10.6%) (****p *<* 0.0001) (Fig. 4B). These findings indicate that in this rat model of PD, DREADD-induced activation of cholinergic neurons at the level of the PPN associates with a substantial increase in SNpc DAergic neuronal activity. This also implicates that the cholinergic–DAergic connectome underlies the clinical effectiveness of PPN-DBS, with the natural downstream target of this neural interaction system being the striatal DAergic neurons that project from the SNpc.

### PPN Cholinergic Activation Results in Contrary Effects on DR1- *Versus* DR2-Bearing MSNs

c-Fos expression was quantified stereologically and expressed as a proportion of the total number of DR1-bearing MSNs of each striatum. MSNs were labeled by DARPP-32 immunostaining (Fig. 4C, D), a “dual-function” DA- and cAMP-regulated phosphoprotein whose phosphorylation at separate threonine residues (34 and 75) has been shown to separately inhibit protein phosphatase-1 and protein kinase A in DAergic neurons [53–55]. DARPP-32 is expressed in both DR1- and DR2-bearing MSNs (in rodents and primates), but not in other GABAergic (inter)neurons of the striatum; hence, it is considered a cellular marker for MSNs [56, 57]. The level of MSNs ipsilateral to the lesioned nigra (with secondary PPN lesion) that co-expressed DR1 or DR2 c-Fos, where animals had been culled either at the CNO OFF or CNO ON stage, was compared with the values for the nonlesioned contralateral side. The mean difference for the percentage DR1-c-Fos MSNs between contralateral and ipsilateral striata in animals during an unstimulated state was deemed negligible, at 14.6 ± 1.1% (*p* *>* 0.05, n.s., *n* = 6) (Fig. 4E). In stimulated animals, the pattern was entirely reversed with higher c-Fos expression seen on the ipsilateral side (91.2 ± 2%) compared with either the intact contralateral side in the same animals (77 ± 4%) (*p *=* 0.0182) or when evaluated against the ipsilateral side of rats in a CNO OFF state (70.7 ± 0.9%) (****p *<* 0.0001, *n* = 6) (Fig. 4E).

The same evaluation performed on DR2-bearing MSNs showed a contrary effect (Fig. 4F); the mean difference in the level of MSNs co-expressing DR2 and c-Fos on the ipsilateral side in unstimulated rodents was 90.1 ± 4.1%, compared with when rats were stimulated (53.4 ± 3.1%) (****p < 0.0001). Taken together, our results reveal that activity within the classically described DR1-mediated “direct” pathway is increased ipsilaterally upon PPN stimulation but reduced on the similar hemispheric side in the DR2-mediated “indirect” pathway. These data provide evidence for a DREADD-dependent reversal of baseline activity imbalance at the striatal level, which in itself appears dependent on nigral DA loss, reflecting classical PD-induced changes in basal ganglia circuitry.

### PPN Cholinergic Stimulation Increases Differential Neuronal Activity Between Thalamic Substructures but Did Not Generate Substantial Overall Thalamic Activation

The neuronal nuclear antigen NeuN was used to immunolabel neurons of the thalamus, which was subsequently separated into the Vl and Vm thalamic subregions, by referring to a stereotaxic atlas [28], its boundaries that were digitally superimposed on matching sections (Fig. 4G). Vl and Vm thalamic subregions were assessed as the PPN sends significant cholinergic efferent projections to these brain regions, with these PPN output regions that associate with regulating several aspects of motor behavior [23, 24]. In lesioned animals, no significant change was noted for mean ipsilateral–contralateral Vl neuronal c-Fos levels, during CNO stimulation (8.2 ± 2%, *n* = 6) compared with the CNO OFF stage (5.3 ± 4.1%) (*p* *=* 0.55, n.s., *n* = 6) (Fig. 4G, H). A similar nonsignificant effect was seen for Vm c-Fos expression (CNO ON 1.1 ± 1.9%; CNO OFF 0.5 ± 4%) (*p* *=* 0.90, n.s.). However, during CNO ON, implying PPN cholinergic activation, Vl thalamic neurons were significantly more activated than Vm ones (**p *=* 0.009) (Fig. 4H).

## Discussion

DREADD are mutated G protein-coupled receptors that are overexpressed within specific neurons, brain regions, or projection pathways. This allows for overexpressed receptors integrated into the neuronal membrane of targeted elements to be silenced or activated in response to the systemic injection of a synthetic agonist for the DREADD, to ultimately permit for dissecting out the neuronal substrate underlying specific behavior. The current study is the first to demonstrate that the combination of 2 powerful techniques, DREADD and *in vivo* PET imaging, can be used with a significant effect to dissect out the functional role of PPN-originating cholinergic pathways, for explaining some of the clinical benefits associated with PPN-DBS to alleviate PD-related disability. By utilizing cholinergic-selective stimulation of remaining PPN neurons in a well-validated rat model of PD, which mirrors both the DAergic nigrostriatal and cholinergic PPN neurodegeneration seen in human PD [10–13], we uniquely demonstrated that the reversal of motor deficits is principally due to PPN cholinergic neurons’ innervation of the nigrostriatal pathway.

We utilized a comprehensive behavioral repertoire to validate the lesion in toxin-lesioned animals, but also to determine the potential of this region-restricted cell-type-specific stimulation paradigm to alleviate PD-related motor deficits. Taken together, the results of the behavioral tests confirm that lactacystin-lesioned rats had sustained significant neuronal loss, to a level sufficient to severely alter motor performance in a variety of tasks. Further supporting this, subsequent postmortem stereological evaluations of nigral TH-ir and PPN ChAT-ir neurons found significant loss of such neuronal groups on the toxin-lesioned brain hemispheric side, compared with the nonlesioned side and also sham-lesioned animals. Future work should concern deeper phenotypic characterization of the motor and neuronal substrate impairments reported here and in our previous work [9–13] for the lactacystin lesion model of PD. In particular, correlations drawn between the different motor function parameters and the number of TH-ir as well as ChaT-ir neuronal cell loss will be insightful for the ongoing characterization of this animal model, with such knowledge that will ultimately provide a crucial contribution for understanding disease mechanisms and guiding subsequent novel therapeutic intervention strategies. In such analyses, future work should not only consider the current combination of measurements, but should also incorporate alternative outcome measures especially those measuring gait disturbance. For such detailed profiling, the behavioral readouts should be correlated with the magnitude of the lesions at several levels. In relation to the dopaminergic lesion, associations should be ascertained between the extent of behavioral impairments and the reduction in dopamine content in the lesioned striatum, the level of lost TH-ir innervation in the ipsilateral striatum, and nigral TH-ir neuronal counts. Similar analyses should determine for the association between the PPN ChAT-ir neuronal counts and various motor outputs; however, as our current results show, deficiency in these and also their rescue are likely to result from the complex interplay between the cholinergic and dopaminergic systems.

SNpc DAergic neurons represent an important output target of the PPN’s cholinergic projecting neurons [58]. When considered during CNO OFF, the c-Fos-based activity analysis showed that loss of DAergic input (secondary to cholinergic excitation) to DR1s within striatal MSNs of lactacysin-induced parkinsonian rats results in ipsilateral striatal DR1 overactivation, with a converse effect that was exerted on DR2s, instead showing downregulated DR2 activity. We propose that this effect forms the mechanistic basis for the profound motor defects shown by unilateral lactacystin-lesioned rats and reflects data from other PET imaging studies that also made use of a D2/3 receptor probe, to reveal that DR1 striatal levels are typically elevated in PD patients prior to DA substitution therapy [59, 60], similar to that seen in other animal models of PD [61]. This phenomenon may be considered a form of denervation supersensitivity that manifests to compensate for diminished DA transmission resulting from the DAergic lesion [62]. On the other hand, in the same rats, the PET-based [^11^C]PHNO displacement analysis during CNO ON evidenced increased striatal DA release, suggesting that the motor recovery shown by the lesioned rats during cholinergic-specific PPN stimulation reflects restored DA imbalance at the striatal level. Immunofluorescence and imaging analysis provided further mechanistic insight into this effect, by revealing that the DA release in the striatum exerted reciprocal effects on the MSNs of the “direct” pathway that predominantly express DR1s, which contrasted to that seen in the “indirect” pathway that mainly express DR2s. Hence, PPN-driven cholinergic overactivation suppressed the activity of striatal DR2-bearing MSNs, but potentiated DR1 MSN activity. Contralateral c-Fos expression was comparable during CNO OFF compared with CNO ON, with the neuronal activity change occurring predominantly ipsilaterally, to strongly suggest that chemogenetic stimulation of the PPN’s cholinergic neurons exerts a powerful downstream effect on striatal MSN activity.

The data underscores the tight regulation that the PPN’s cholinergic systems exert over DA transmission at the level of cell bodies based in the midbrain. In addition to its connections with the basal ganglia, the PPN also sends dense neuronal projections to the thalamus, a high proportion of these being cholinergic [63]. Compared with the results we obtained for nigral and striatal neuronal activation, our study shows that PPN cholinergic input at the levels of the Vm and Vl thalami might only play a minimal role in mediating the motor behavioral improvements seen in this rat model of PD, upon selective activation of PPN cholinergic neurons. No significant change was detected between CNO ON compared with the CNO OFF stage when mean ipsilateral–contralateral neuronal c-Fos levels of either the Vl or ML were compared in lesioned rats; however, a significant upregulatory effect was seen for the Vl when compared with the Vm during CNO ON. These findings warrant further investigation in future work, with much relating to the structural–functional interactions between the thalamus, basal ganglia, and PPN that remain to be ascertained. For instance, output pathways of the Vm thalamic nucleus was suggested to rapidly compensate for functional impairments affecting the Vm [64]. Hence, it remains possible that the relatively low levels of neuronal activity seen in the Vm of parkinsonian rats, and also the lack of significant neuronal activity in the Vm following PPN cholinergic activation, could be due to such compensatory changes.

The thalamus receives both direct input from the PPN [23, 24], as well as indirect input from the PPN via the striatum, and could therefore rather be seen as lying further downstream of the striatum, showing neuronal activity in consequence to striatal modulation [65]. This view complicates the interpretation of the present results. Furthermore, at a single-neuronal level, understanding still lacks as to how information conveyed via neurons from the PPN to the thalamus is integrated into motor control areas. Such complex reciprocal interconnections between the thalamus and PPN, but also between thalamic neurons and another of the PPN cholinergic neurons’ target, the striatum, suggest for complex patterns of activity within the thalamus as a result of various inputs, with definitive assertations as to the complex interplay of thalamic-related feedback pathways involved in the behavioral effects seen here that lie outside the scope of the current study. Future work should utilize tools that complement the Fos-based approach we used here, such as retrograde tracing techniques using viral vectors for reconstructing axonal arborization and *in vivo* electrophysiology recordings by which to map and subsequently assess thalamic regional differences in neuronal activation levels, in the presence and absence of PPN cholinergic-specific excitation.

Taken together, our results showing heightened activation in both the striatum and SNpc following PPN cholinergic-specific activation reinforce the notion that the pedunculopontine–nigrostriatal pathway is the most likely neural pathways underlying the behavioral recovery seen in this rat model of PD. However, the source of the DA release is presently unknown, where the current study did not directly allow for a distinction to be drawn between the degree to which the striatum *versus* the SNpc might provide a functional connection with the PPN that is sufficient to evoke the behavioral recovery seen in this animal model of PD. Future work should fully dissect out the relative functional contributions made by the various neural structures encompassing the nigrostriatal dopaminergic pathway, toward the improved motor function reported here. Identifying the involvement of a neuronal pathway in specific behavior is challenging due to the large number of synaptic connections in the brain. Here we described experiments involving DREADD-based stimulation of neuronal cell bodies, and hence, the present study did not directly address the effects at the axonal ends. The current data contributes to guiding future work aimed at comparing output measures resulting from application of pathway-based neuromodulatory tools to PPN neurons projecting to either the SNpc of the striatum, with a large body of literature that supports the presence of such distinct PPN output pathways [6, 21, 22, 25]. Experimental efforts to interrogate the various components of this functional network could lead to clinically exploitable substructural targets for improving PPN-DBS delivery in a clinical context.

Commonly used treatments for PD are only partially or transiently effective. Our current findings support the notion that the most effective therapies for PD should target *both* the DA and ACh modulatory systems. Based on our current findings, we propose a model whereby increasing cholinergic control of DA transmission to modify DR1 and DR2 signaling in the striatum could alleviate PD-related motor dysfunction. Future work should determine whether modifying the signaling levels of DR1 and DR2s in this manner could also protect nigrostriatal DA neurons from toxic damage. Stimulating DA release from residual DA terminals by means of nicotinic acetylcholine receptor agonists has already been highlighted as a potentially effective treatment for PD [66]. Our study suggests the enhancement of hM3 receptor activity as a candidate drug strategy for disorders involving the DA system including PD, for potentiating striatal DAergic signaling. In this regard, a region-specific increase in muscarinic acetylcholine receptors (mAChRs) seen in PD postmortem brains has been described as a compensatory mechanism that decelerated the development of cognitive symptoms [67]. To validate the therapeutic viability of this approach, our results will require further elaboration with the use of subtype-specific mAChR antagonists. In support, earlier work has highlighted the therapeutic potential of muscarinic antagonists against PD [68]. However, the relative expression of different mAChRs and how such density changes could underlie PD symptoms remains underexplored, with such knowledge that could form the basis for developing more selective ligands for the muscarinic receptors, hence allowing more targeted approaches for treating PD. Possibilities include the use of selective allosteric activators at specific mAChRs for redressing the imbalance in striatal DA levels in PD-affected brains. This strategy has shown effectiveness, by avoiding detrimental effects on cognition seen with the use of nonselective mAChRs antagonists [68].

Clinical evidence in support of an ACh–DA interaction in the brain includes the finding that degeneration of SNc DAergic and cholinergic neurons in the nucleus basalis of Meynert [69] and PPN [15] concurrently commences during Braak stage III. The role of PPN cholinergic neurons in anatomical studies, which has more recently translated into a focus placed on their functions, remains an active and evolving topic in neuroscience. However, balancing the intricate interaction at play between these neuromodulatory systems through the use of pharmacological tools has proved troublesome in a clinical context; for instance, use of the DA replacement agent L-DOPA or DAergic agonist drugs was shown to increase the risk for patients developing cognitive symptoms, regarded as stemming from cholinergic neuronal loss or dysfunction, following prolonged drug use [70, 71]. In this regard, our study highlights the need for neuromodulatory therapies to consider the intricate interaction between the ACh and DA neuromodulatory systems, as pharmacological amelioration of specific aspects of PD, modulated by either the DA/ACh system, was shown to bring undesirable side effects through counterproductive effects on the other system [72]. Such contrasting effects by DA- *versus* ACh-mediated therapies make it crucial that a better understanding be reached as to DA/ACh interaction at the molecular and circuit levels, for designing novel and more specific treatments capable of avoiding such undesirable side effects.

Hence, the current study correlated structural connectivity between PPN cholinergic neurons and nigrostriatal DAergic ones to ACh–DA functional connectivity, the activation of which was sufficient to eliminate motor dysfunction in the lactacystin-induced rat model of PD. Our findings provide a contextual framework for such interactions by demonstrating how the upregulating activity of the PPN cholinergic system directly modulates the nigrostriatal DA one, to form the mechanistic basis of the remarkable recovery in motor function shown by the parkinsonian rats after receiving PPN cholinergic stimulation.

## Conclusions and Future Outlook

Our analysis of the activity levels of neurons comprising the “direct” pathway as well as those of the “indirect” pathway [73] raises several intriguing possibilities for how cholinergic-specific targeting of PPN somas could achieve more effective alleviation of the axial-related motor dysfunctions seen in PD, than through the use of conventional DBS that does not offer cell-type-specific modulation, but rather produces global stimulation of the brain nucleus without discerning between different cell types, thus potentiating undesirable off-target effects. Because DBS nonselectively modulates local neuronal activity, it is difficult to tell which of its various network effects induce therapeutic outcomes and which might cause side effects. Studies such as ours that investigate the functional basis of DBS provide information to allow for designing more effective anti-parkinsonian treatments. In particular, the current data could allow for designing medical approaches capable of improving the ratio between desirable and undesirable outcomes and leaving nonimpaired functions intact. For example, specific genetically defined neurons within precise subcircuits could be targeted to treat motor symptoms of PD, without inducing a cognitive detriment, and vice versa. In this regard, the feasibility for innovative site-specific DBS approaches combined with pharmacological tools has already been demonstrated [73].

Current DREADD-based neuromodulatory tools are suitable for human CNS disease applications. However, improved AAV-mediated gene delivery methods [74] as well as development of novel, highly selective DREADD agonists [75] offer clear promise for the extension of DREADD-based neurotherapeutics to human patients. The promise such novel clinical strategies hold can only be met if guided by studies such as the current report, aimed at identifying disease-relevant neuro-types and subcircuits for therapeutic targeting. Given the pressing need for developing new, rational therapies for PD, the development of circuit and cell-type-specific pharmacological compounds should be considered in designing optimally targeted therapies.

### Limitations

As with the majority of experimental work, some limitations should be noted for the current study, to be addressed in future research. Firstly, a translational investigative approach, namely PET imaging, was used to measure *in vivo* changes in DAergic signal. Future preclinical studies using more direct measures of neurotransmitter release with improved temporal resolution, such as microdialysis or fast cyclic voltammetry, will allow for discriminating the signal further. Moreover, some restrictions relating to the presently used c-Fos, as a reporter of neuronal activation, have been reported, including low temporal resolution [76–78]. However, c-Fos mRNA or its protein product remains 1 of the most reliable cellular markers by which to identify neuronal activation in the brain [40, 78–80], with Fos-based approaches utilizing immunohistochemical reporters that are particularly suited for application to neurons of interest residing within deep brain nuclei, such as the neuronal structures that were studied here. To expand on the present findings, future studies should include alternative technologies relative to a c-Fos reporter assay, i.e., by correlating the increased c-Fos activity in the striatum and SNpc following DREADD-based PPN cholinergic activation with electrophysiological activity recordings made in these brain structures.

Lastly, recent rodent-based studies showed that CNO is not completely inert, but reverse-metabolizes to clozapine, which can cross the blood–brain barrier to cause its own CNS effects [81, 82]. However, this view has been challenged by other works, including reporting no significant retroreduction of CNO to clozapine in mice [30], whereas Jann and others [83] reported no detectable clozapine after CNO administration to rats. In addition, formation of clozapine from CNO was shown to be inhibited to ascorbate [84] that is synthesized endogeneously in rodents [85], suggesting that only limited CNO retroconversion might be occurring in experimental rodents. Here and also previously [12], we show that the cellular and behavioral effects induced via PPN-restricted, cholinergic-specific DREADD excitation are not due to clozapine binding. Using the same animal model as used here, we transfected PPN cholinergic neurons with a fluorescently tagged “control” viral vector, void of the DREADD construct. Cellular and behavioral effects were compared with those induced by the activating DREADD-containing viral vector. Because cells expressing this control construct (hChR2(H134R)-eYFP) are activated optogenetically [86], but remain unresponsive to CNO, the strategy allowed for validating that the observed effects resulted from the DREADD ligand only. Moreover, we restricted toxin/sham (intra-SNpc) and active DREADD (intra-PPN) to 1 cerebral hemisphere only, with the noninjected brain hemisphere serving as internal control, allowing for ipsilateral–contralateral comparisons across the midline, e.g., for MSN DR1 and DR2 activation status, [^11^C]PHNO BP_ND_ and rats’ behavioral performance in tests relying on cross-midline forepaw placing [35]. In light of recent debate relating to the potential non-DREADD-mediated effects of CNO, future studies aiming to expand on the current results should include adequate control conditions to demonstrate that the observed effects are not due to binding of CNO’s parent compound, clozapine. It is also worth considering the reports that direct intracranial CNO administration within a target region may avoid retroconversion of CNO to clozapine [87], whereas CNO delivered intracerebroventricularly might yield more consistent results compared with systemic CNO administration [88].

## Electronic supplementary material

ESM 1 (PDF 3427 kb).

## Data Availability

All research data presented in the current paper represents original research data newly acquired by the authors listed. Should the animal-related raw data be required, the corresponding author can be contacted for a copy. However, in accordance with Imperial College London data sharing policy, any person requesting such data, and the institution they are affiliated with, will be asked to agree to a formal data sharing agreement before the data will be sent. Matlab codes used for computationally analyzing the PET data shown here in summary form should be requested from the corresponding author who will request this from the author of this code, Dr. Lisa Wells (Invicro). Under the conditions of Invicro company policy, those requesting such code will be asked to agree to a formal data sharing agreement.

## Abbreviations

*ACh*: acetylcholine
*AAV*: adeno-associated viral vector
*AP*: anterior–posterior
*asf*: area sampling fraction
*BP*_*ND*_: binding potential
*ChAT*: choline acetyltransferase
*CNO*: clozapine *N*-oxide
*CT*: computed tomography
*DA*: dopamine
*DAergic*: dopaminergic
*DMSO*: dimethyl sulfoxide
*DARPP-32*: dopamine- and cAMP-regulated phosphoprotein
*DR*: dopamine receptor
*DR1*: dopamine receptor class-1 receptor
*DR2*: dopamine receptor class-2 receptor
*DREADD*: designer receptors exclusively activated by designer drugs
*EtOH*: ethanol
*eYFP*: enhanced yellow fluorescent protein
*hM3*: human M3 muscarinic
*hsf*: height sampling fraction
*ir*: immunoreactive
*i.p*: intraperitoneally
*L:R*: left:right
*mAChRs*: muscarinic acetylcholine receptors
*ML*: medio-lateral
*MSNs*: medium spiny neurons
*OF*: open field
*PET*: positron emission tomography
*PFA*: paraformaldehyde
*PHNO*: 4-propyl-3,4,4a,5,6,10b-hexahydro-2H-naphtho[1,2-b][1,4]oxazin-9-ol
*PIT*: postural instability test
*PPN*: pedunculopontine nucleus
*ROI*: regions of interest, *rpm* rotations/min
*RT*: room temperature
*SNpc*: substantia nigra pars compacta
*SEM*: standard error of the mean
*SRTM*: simplified reference tissue model
*ssf*: section sampling fraction
*SUVs*: standardized uptake values
*TACs*: time–activity curves
*TBS*: Tris buffered saline
*TH*: tyrosine hydroxylase
*VEFP*: vibrissae-evoked forelimb placement
*Vl*: ventrolateral
*Vm*: ventromedial
*WT*: wild type
